# RHBDD1 upregulates EGFR via the AP-1 pathway in colorectal cancer

**DOI:** 10.18632/oncotarget.15694

**Published:** 2017-02-25

**Authors:** Fei Miao, Mengmeng Zhang, Yuechao Zhao, Xiaolu Li, Rongyan Yao, Fan Wu, Rong Huang, Kai Li, Shiying Miao, Changwu Ma, Hongge Ju, Wei Song, Linfang Wang

**Affiliations:** ^1^ Department of Biochemistry and Molecular Biology, State Key Laboratory of Medical Molecular Biology, Institute of Basic Medical Sciences Chinese Academy of Medical Sciences, Peking Union Medical College, Beijing 100005, China; ^2^ Department of Pathology, Baotou Medical College, Baotou 014040, China; ^3^ Department of Pathology, The First Affiliated Hospital of Baotou Medical College, Baotou 014010, China; ^4^ Department of Medical Oncology, Chifeng Municipal Hospital, Chifeng 024000, China

**Keywords:** colorectal cancer, EGFR, RHBDD1, AP-1, c-Jun

## Abstract

Our previous study showed that RHBDD1 can activate the EGFR signaling pathway to promote colorectal cancer growth. In the present study, EGFR was decreased when RHBDD1 was knocked down or inactivated. Further analysis found that c-Jun and EGFR protein expression was decreased in RHBDD1 knockdown and inactivated cells. c-Jun overexpression in RHBDD1-inactivated cells rescued EGFR expression in a dose-dependent manner. RHBDD1 overexpression in RHBDD1-inactivated cells restored EGFR expression, but this effect was counteracted by c-Jun knockdown. Furthermore, EGFR and c-Jun were attenuated in the RHBDD1 knockdown and inactivated groups in animal tumor models. Tissue microarray assays demonstrated a correlation between RHBDD1 and EGFR in colorectal cancer patients. Therefore, our findings indicate that RHBDD1 stimulates EGFR expression by promoting the AP-1 pathway.

## INTRODUCTION

Colorectal cancer (CRC) is one of the most commonly diagnosed cancers worldwide, ranking third in men and fourth in women with nearly 1.2 million new cases expected each year [1, 2]. Epidermal growth factor receptor (EGFR) is a member of the ErbB family, which includes EGFR (also known as ErbB1/HER-1), ErbB2/Neu/HER-2, ErbB3/HER-3, and ErbB4/HER-4, and is a type of receptor tyrosine kinase located in the membrane. Binding of ligands, such as epidermal growth factor (EGF) and transforming growth factor alpha (TGF-α), induces homo- or heterodimerization and receptor activation, subsequently activating downstream signaling pathways, including the extracellular-signal-regulated kinase (ERK) and phosphatidylinositol 3-kinase (PI3K)/protein kinase B (Akt) pathways. These pathways regulate several cellular processes, such as proliferation, apoptosis, migration and angiogenesis [3]. EGFR is normally expressed in many cell types, including epithelial and mesenchymal lineages [4]. EGFR overexpression has been observed in many cancers, such as head and neck cancer, pancreatic cancer, and colorectal cancer [5–7]. A previous study showed that EGFR is highly overexpressed in 25%-82% of CRC patients [8], and EGFR expression plays a pivotal role in the prognosis or survival of CRC patients [9–12].

Rhomboid family proteins are intramembrane serine proteases that are highly conserved in many species [13]. Previous studies showed that rhomboid proteases cleaved transmembrane proteins via a catalytic histidine–serine dyad in the polytopic rhomboid core domain [14–17]. Rhomboid-1, the best-characterized family member, cleaves the EGFR ligand Spitz to activate EGFR signaling in *Drosophila* [18, 19]. However, several rhomboid proteins have lost their proteolytic activity and are inactive rhomboids called rhomboid pseudoproteases, which include derlins and iRhoms [20, 21]. These inactive rhomboids function by binding substrates in the eukaryotic secretory pathway and regulating their trafficking or degradation. iRhom2 can facilitate ADAM17 cleavage of TGF-α by transporting ADAM17 from the endoplasmic reticulum to the Golgi complex [22, 23]. A previous study reported that RHBDL2 can activate the mammalian EGF receptor [24], and we found that RHBDD1 can cleave proTGF-α, releasing active ligands and therefore enhancing the EGFR signaling pathway [25]. Recent research has implicated Rhomboid proteins in cancers. A prior report showed that RHBDF1 expression is highly elevated in breast cancer and strongly correlated with increased disease progression, metastasis, poor prognosis, and poor response to chemotherapy [26]. RHBDD2 mRNA and protein are overexpressed in breast cancer [27]. Based on these results, we propose that RHBDD1, a member of Rhomboids, may play a role in colorectal cancer by interacting with EGFR.

In the present study, we investigated the role of RHBDD1 on EGFR in colorectal cancer. We found that RHBDD1 activates c-Jun, which in turn activates EGFR expression. Therefore, RHBDD1 may be useful in colorectal cancer therapy as a therapeutic target in combination with EGFR antibodies.

## RESULTS

### RHBDD1 silencing decreases EGFR protein expression

**Figure 1:**
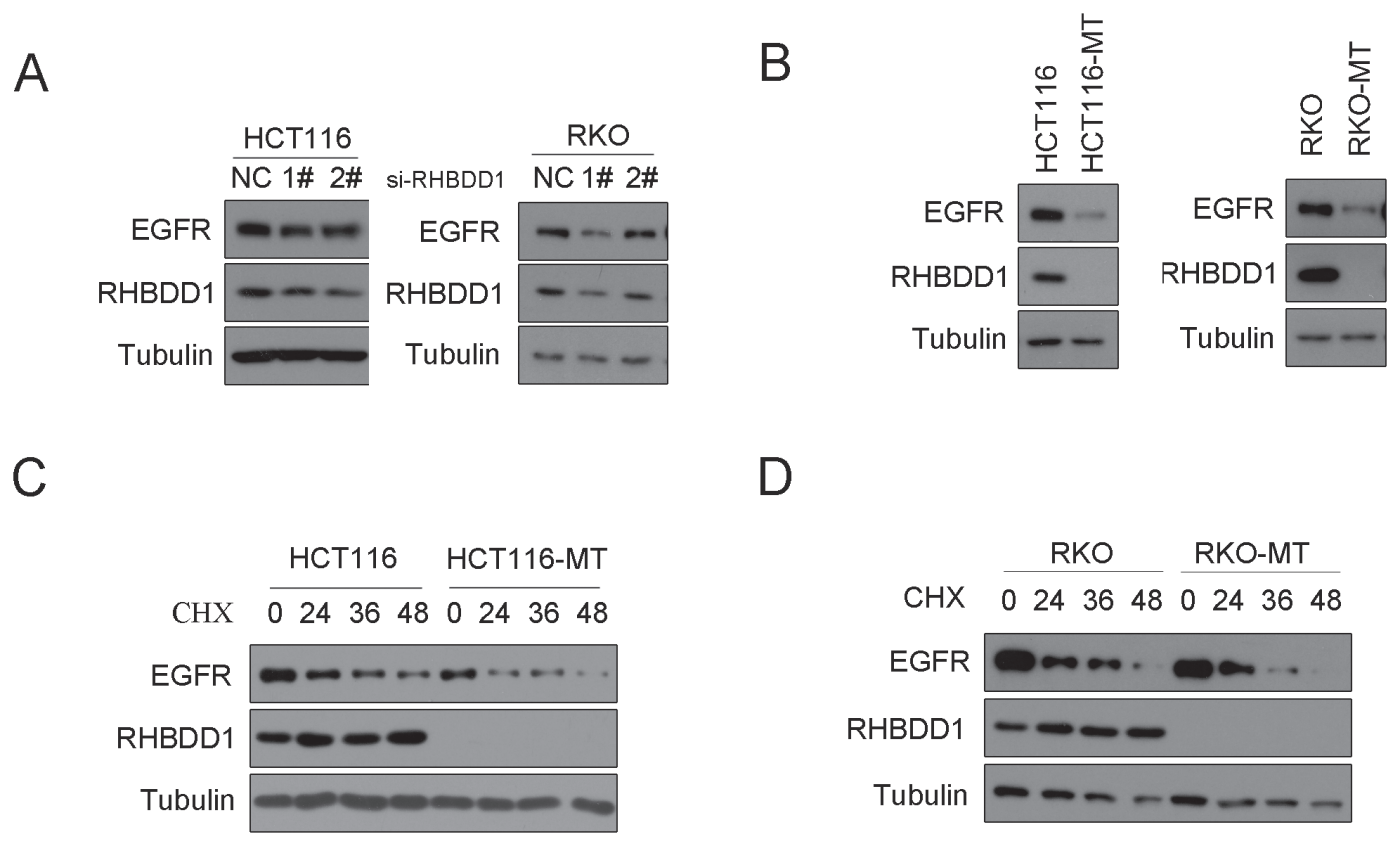
RHBDD1 attenuation decreases EGFR protein expression **A**. RHBDD1 knockdown reduces EGFR protein expression. RHBDD1-shRNA plasmid and a negative control were transfected into RKO and HCT116 cells. After 24 h, the cells were extracted for Western blot analysis using the indicated antibodies. **B**. RHBDD1 knockout can attenuate EGFR protein expression. RKO, RKO-MT, HCT116 and HCT116-MT cells were extracted for Western blot analysis using the indicated antibodies. **C, D**. RHBDD1 inactivation decreases EGFR protein stability. EGFR protein was detected at 0 h, 24 h, 36 h and 48 h after chlorhexidine treatment in RKO, HCT116 and the RHBDD1-inactivated cells.

To determine whether RHBDD1 stimulates EGFR, we assessed EGFR expression following RHBDD1 knockdown by Western blot analysis. We transfected siRNAs into HCT116 and RKO cells, and after 48 h, we measured EGFR expression. As shown in Figure 1A, EGFR expression decreased following RHBDD1 silencing in both HCT116 and RKO cells. To further confirm these results, we observed EGFR expression in RHBDD1-inactivated HCT116 and RKO (HCT116-MT, RKO-MT) cells. These RHBDD1-inactivated cells were constructed using a somatic cell knock-in method [25]. RHBDD1 protein was not detected by Western blotting in the RHBDD1-inactivated cells. EGFR expression was markedly decreased in both RHBDD1-inactivated cells (Figure 1B). Then, we used cycloheximide (CHX) to inhibit protein synthesis to determine whether RHBDD1 had an effect on EGFR stability. After addition of CHX to the HCT116-MT cell culture medium, cells were harvested at 0 h, 24 h, 36 h and 48 h. EGFR protein was detected and showed accelerated degradation in the RHBDD1-inactivated cells (Figure 1C). We then observed EGFR protein stability in RKO and RKO-MT cells. Treatment with CHX led to more rapid degradation of EGFR in the RHBDD1-inactivated cells.

### RHBDD1 silencing decreases EGFR mRNA levels

**Figure 2:**
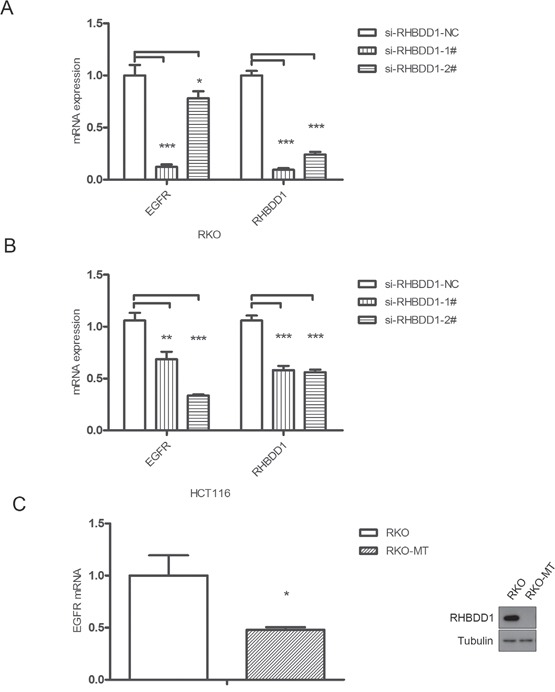
RHBDD1 silencing reduces EGFR mRNA expression **A**. Transient knockdown of RHBDD1 attenuated EGFR mRNA expression. Two RHBDD1 siRNAs and a negative control were transfected into RKO cells, and mRNA was analyzed by real-time qPCR after 48 h. **B**. RHBDD1 stable knockdown attenuates EGFR mRNA expression. EGFR mRNA was detected in HCT116 RHBDD1 stable knockdown cell lines. **C**. RHBDD1 inactivation decreases EGFR mRNA expression. EGFR mRNA was observed in RKO and RHBDD1-inactivated RKO cell lines. The results are shown as a bar graph. The data are representative of three different experiments, and the error bars represent the standard deviations of triplicate samples. (mean±SEM, Student's two-tailed t-test, *P<0.05, **P<0.01, ***P<0.001).

After demonstrating that RHBDD1 can stimulate EGFR protein expression, we hypothesized that RHBDD1 may increase EGFR mRNA. To test this hypothesis, we transfected si-RHBDD1-1#, si-RHBDD1-2# and a negative control into RKO cells. After 48 h, we measured EGFR mRNA levels using real-time PCR. The results demonstrated that RHBDD1 knockdown significantly attenuated EGFR mRNA levels (Figure 2A). Then, we observed EGFR mRNA levels in HCT116 cells with stable RHBDD1 knockdown (HCT116-sh) and control cells (HCT116-con). As shown in Figure 2B, EGFR mRNA levels was notably decreased when RHBDD1 was stably knocked down. To further confirm that RHBDD1 could increase EGFR mRNA levels, we performed real-time PCR using RKO-MT and RKO cells. As expected, EGFR mRNA levels significantly decreased following RHBDD1 inactivation (Figure 2C). Therefore, we concluded that RHBDD1 positively stimulates EGFR mRNA levels.

### RHBDD1 stimulates EGFR via c-Jun

**Figure 3:**
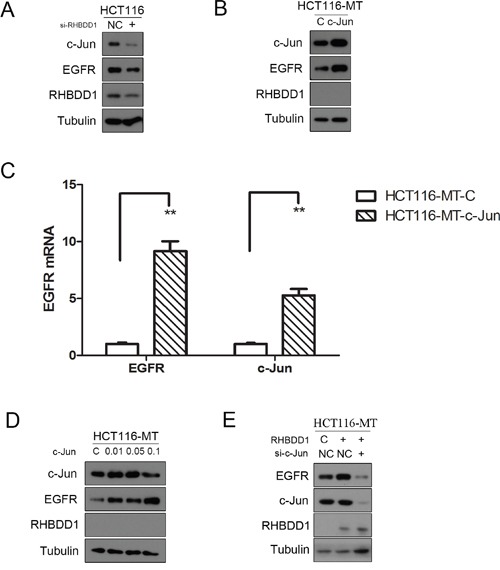
RHBDD1 stimulates EGFR expression through c-Jun **A**. RHBDD1 silencing attenuates EGFR and c-Jun expression. HCT116 cells were transfected with RHBDD1 siRNA and negative control. The cells were extracted after 48 h for Western blot analysis using the indicated antibodies. **B, C**. Heterogeneous expression of c-Jun rescued EGFR expression in RHBDD1 inactivation cells. HCT116 cells were transfected with a c-Jun-expressing plasmid and the control vector. The cells were analyzed by Western blot and real-time qPCR analysis (the data are representative of three different experiments, mean±SEM, Student's two-tailed t-test, **P<0.01) after 24 h. **D**. c-Jun increased the expression of EGFR in a dose-dependent manner. HCT116-MT cells were transfected with different amounts of c-Jun. EGFR protein was measured. **E**. RHBDD1 rescued EGFR expression and was inhibited by c-Jun downregulation. HCT116-MT cells were transfected with a RHBDD1-expressing plasmid and the control vector plus c-Jun siRNA or the negative control.

Our previous study showed that RHBDD1 can positively activate c-Jun expression [28]. Other reports have shown that c-Jun increases EGFR mRNA expression [29, 30]. Taken together, these results suggested that RHBDD1 may regulate EGFR via c-Jun. We first assessed EGFR and c-Jun protein expression when RHBDD1 was knocked down in HCT116 cells. Both EGFR and c-Jun protein expression declined following RHBDD1 knockdown (Figure 3A). We then transfected the c-Jun vector and control vector into HCT116-MT cells. Cells were harvested after 24 h. EGFR mRNA and protein were detected by real-time PCR and Western blot, respectively. The results are shown in Figure 3B, 3C, and both EGFR protein and mRNA were elevated after c-Jun overexpression. We further tested whether c-Jun overexpression had a dose-dependent effect. We transfected 0.01 μg, 0.05 μg, and 0.1 μg c-Jun vector and control vector into HCT116-MT cells. EGFR protein expression was measured after 24 h. As shown in Figure 3D, c-Jun enhanced EGFR protein expression in a dose-dependent manner. RHBDD1 was re-expressed in the HCT116-MT cell lines, and subsequently, c-Jun was knocked down. The results are shown in Figure 3E. EGFR expression was increased with RHBDD1 re-expression but was further suppressed when c-Jun was subsequently knocked down.

### RHBDD1 knockdown/inactivation attenuates EGFR and c-Jun protein expression *in vivo*

**Figure 4:**
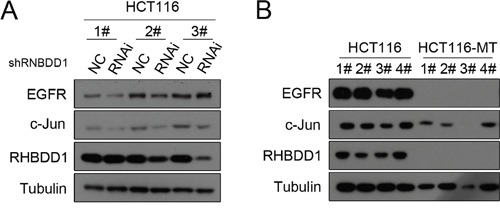
The effect of RHBDD1 on EGFR and c-Jun expression *in vivo* **A**. Stable knockdown of RHBDD1 decreases EGFR and c-Jun expression. HCT116 stable knockdown cells and control cells were subcutaneously injected into nude mice, and tumors were removed and analyzed by Western blotting after 21 d using the indicated antibodies. **B**. RHBDD1 inactivation attenuates EGFR and c-Jun expression. HCT116 and HCT116-MT cells were treated using the methods described in A.

To verify that RHBDD1 could stimulate EGFR through c-Jun *in vivo*, colorectal cancer cells were subcutaneously injected into BALB/c nude mice to determine the effect of RHBDD1 KD or KO on EGFR and c-Jun protein expression. We first conducted the experiments using HCT116-shcon and HCT116-shRHBDD1, and after 20 days, tumor tissues were detected with Western blot analysis. As shown in Figure 4A, EGFR protein expression was decreased in the RHBDD1 knockdown group, as well as the c-Jun protein. We then used RHBDD-inactivated HCT116 and HCT116 cells to perform the same subcutaneous tumor experiments. Using equivalent treatments, we found that EGFR and c-Jun protein expressions were both dramatically attenuated (Figure 4B).

### RHBDD1 and EGFR expression in colorectal cancer patients

**Figure 5:**
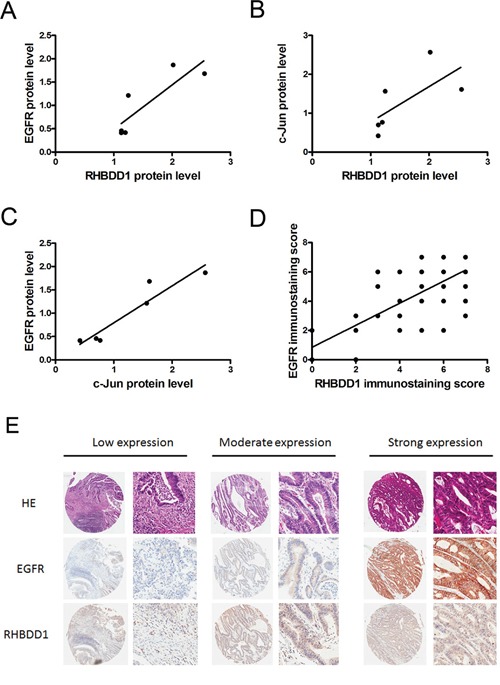
RHBDD1 and EGFR expression in colorectal cancer **A, B, C**. Expression of RHBDD1, c-Jun and EGFR in CRC patients was analyzed by Western blotting using Tubulin as a loading control. The level of RHBDD1 expression was plotted against the level of EGFR and c-Jun, and the level of c-Jun expression was plotted against the level of EGFR. **D**. RHBDD1 expression was positively correlated with EGFR expression in tissue microarray analysis (Spearman rank correlation, P<0.01). **E**. Representative RHBDD1 and EGFR expression in CRC tissues detected by tissue microarray staining (magnification×35, ×200).

Our lab previously found that RHBDD1 is overexpressed in colorectal cancer tumors [25], and EGFR was often overexpressed in many cancers [6, 7, 25]. Therefore, we assessed the correlation between RHBDD1 and EGFR in colorectal cancer patient samples. First, we detected the protein expression in colorectal cancer patient samples (Supplementary Figure 1). The relative protein level of RHBDD1 was plotted against EGFR and c-Jun, separately, and c-Jun was plotted against EGFR (Figure 5A, 5B and 5C). Statistical analysis showed Spearman's correlation coefficients of 0.886, 0.943 and 0.943 with associated P value 0.033, 0.017 and 0.017, indicating a significant positive correlation between either two of RHBDD1, c-Jun and EGFR. We further conducted experiments to confirm the results. A tissue microarray was then used to determine RHBDD1 and EGFR expression in colorectal cancer. Spearman's analysis showed strong positive correlations between RHBDD1 and EGFR, with a coefficient of 0.62 (Figure 5D). Our data are shown in Table 1. RHBDD1 and EGFR were correlated regardless of the pathological parameters, including age, gender, tumor size, tumor grade and TNM stage. The correlation coefficient, R, was greater than 0.5, showing a strong positive relationship between the two proteins, except for the TNM stage 1-2 group, which was 0.471. There were no notable differences within gender and tumor size groups. For tumor grade, the R value was much higher in the II-III/III group, indicating that RHBDD1 and EGFR expression was significantly associated with advanced-stage tumor grade. Furthermore, the RHBDD1 and EGFR correlation was stronger in late-stage colorectal cancer patients. As shown in Figure 5E, lower expression of RHBDD1 expression was found with lower expression of EGFR, with a similar pattern for moderate expression and high expression.

**Table 1:** EGFR and RHBDD1 correlation characteristics in colorectal cancer

Clinicopathologic parameters	N	R^a^ Value(EGFR and RHBDD1 expression correlation)
All cases	74	0.620
Age		
<65	34	0.715
≥65	40	0.532
Sex		
Male	42	0.593
Female	32	0.677
Size		
<5cm	31	0.570
≥5cm	43	0.659
Grade		
I-II/II	61	0.562
II-III/III	13	0.860
TNM Stage		
1-2	36	0.471^b^
3-4	38	0.717

^a^ R Value are obtained from Spearman analysis, P<0.01; b P<0.05.

## DISCUSSION

Rhomboid family proteins are known to cleave membrane proteins in the membrane catalytic site, releasing protein domains that contribute to the functional activation of substrates that control a wide variety of biological processes. The catalytically inactive rhomboid-like proteins, such as iRhoms and derlins, regulate membrane proteins by interacting with the substrate to prevent their cleavage. EGFR and its downstream signaling pathway have been associated with rhomboids. Rhomboid-1 activates EGFR signaling by cleaving the EGFR ligand Spitz in *Drosophila* [18, 19]. RHBDL2 cleaves EGF, thereby facilitating its secretion and triggering activation of the EGFR [31]. iRhoms 1 and 2 are crucial upstream regulators of ADAM17-dependent EGFR signaling [32]. Our recent work demonstrated that RHBDD1 could cleave proTGF-α to activate the EGFR signaling pathway. These studies associating rhomboids with EGFR predominantly focused on cleavage of its ligands and subsequent activation of its downstream signaling pathway. However, little is known about whether rhomboids could directly stimulate EGFR expression. Our study shows for the first time that a rhomboid protein can stimulate EGFR expression.

In this study, we first found decreased expression of the EGFR protein following RHBDD1 knockdown in HCT116 and RKO colorectal cancer cell lines. With further confirmation in RHBDD1-inactivated cells and protein stability experiments, we concluded that RHBDD1 can increase the EGFR protein level. However, the underlying mechanism remains unknown. Protein stability experiments showed that RHBDD1 may stabilize the EGFR protein by inhibiting degradation. Although EGFR showed increased degradation in RHBDD1-inactivated cells, it may be because EGFR protein was already lower in these cells than that in the wild type. These results should be confirmed in further experiments. We speculated that this regulation may occur at mRNA level because mRNA directly regulates protein expression.

After demonstrating that RHBDD1 increased EGFR mRNA, we further investigated the potential link between RHBDD1 and EGFR. AP-1 can stimulate EGFR mRNA expression [29]. AP-1 binds 7 different sites in the EGFR promoter, and heterogeneous expression of c-Jun promotes EGFR expression [30]. Our previous work demonstrated that RHBDD1 positively increases c-Jun expression [28]. Taken together, these results establish the RHBDD1-c-Jun-EGFR axis. Two major questions need to be answered: can c-Jun rescue RHBDD1 inactivation caused by EGFR downregulation? Can c-Jun silencing decrease EGFR expression after RHBDD1 rescue in RHBDD1-inactivated cells? We further confirmed that EGFR expression was elevated at both the protein and mRNA levels, and it was restored when RHBDD1 was re-expressed and further decreased when c-Jun was knocked down. These results suggest that RHBDD1 positively stimulates EGFR by regulating c-Jun expression.

RHBDD1 is overexpressed in CRC [25], and EGFR is overexpressed in many cancers [12, 33–37]. Previous data have demonstrated the RHBDD1 positively stimulates EGFR, and we then observed a correlation between RHBDD1 and EGFR in CRC patients. The data showed that RHBDD1 is strongly positively correlated with EGFR. Regardless of any pathological parameters, RHBDD1 was correlated with EGFR, but in advanced-stage tumors and late-stage patients, their correlation was much stronger, indicating that they may play an important role in advanced-stage tumors. Because EGFR has a vital role in cancer development, many studies have focused on anti-EGFR treatment. Numerous reports have shown that anti-EGFR treatment can inhibit tumor cell survival, growth, proliferation and differentiation [38–40]. However, in the presence of K-RAS, B-RAF and other gene mutations, the anti-EGFR treatments lose their efficacy [41–44]. Targeting RHBDD1, possibly through inactivation, can decrease EGFR expression and its downstream signaling pathways even in the presence of K-RAS or B-RAF mutation. HCT116 possesses K-RAS mutations and RKO possesses B-RAF mutations, but inactivation of RHBDD1 in these cells resulted in decreased EGFR expression and inactivation of the downstream signaling pathway, which is the major reason why anti-EGFR treatments fail ([25], RKO data in Supplementary Figure 2). By using small molecules, siRNAs, or RNA aptamers [45] or inhibitors targeting RHBDD1 may be another potential method to treat cancers.

In summary, our results showed that EGFR can stimulated by RHBDD1 by positively regulating c-Jun expression. We showed for the first time that RHBDD1, a member of the rhomboid family proteins, is involved in regulating EGFR expression. This report provides new insights into CRC therapy, EGFR therapy and a potential new therapeutic target.

## MATERIALS AND METHODS

Cell lines were obtained from the Cell Source Center of the Institute of Basic Medical Sciences, Chinese Academy of Medical Sciences. HCT116 and RKO cells were cultured in IMDM (HyClone, Thermo Scientific) with 10% FBS in 5% CO_2_ incubators at 37 °C. Cells were passaged every 2–3 days with 0.5 mg/ml trypsin (1:250) and 0.53 mM ethylenediaminetetraacetic acid (EDTA). The expression plasmid for c-Jun was pcDNA6.0. The sequences of the two RNAi oligos targeting RHBDD1 and one c-Jun RNAi oligos were GUAGAUGGUUUGCCUAUGUTT, GGAUUCUUGUUGGACUAAUTT and GCUGAUUACUGUCAAUAAATT(purchased from GenePharma). Chlorhexidine (CHX) was purchased from Sigma-Aldrich.

### Antibodies

The RHBDD1 mouse monoclonal antibody was prepared in our laboratory. Other antibodies used in this study were anti-EGFR (Cell Signaling Technology), anti-c-Jun (Cell Signaling Technology), and anti-tubulin (Sigma-Aldrich).

### Western blot

Cells were washed with PBS, harvested, lysed with SDS lysis buffer (50 mM Tris–HCl [pH 6.8], 10% glycerol, and 2% SDS), and then quantified using BCA protein assay reagent (Pierce). The extracts were separated using 10% SDS-PAGE and were then electrophoretically transferred to a PVDF membrane (GE Healthcare) according to standard protocols. The membrane was blocked in 5% skim milk for 1 h at room temperature and then incubated overnight with the indicated antibodies at 4 °C.

### RNA isolation and RT-PCR and real-time PCR

Total RNA was isolated from the cultured CRC cell lines and tumors by TRIzol reagent (Invitrogen). cDNA synthesis was performed using same amount of RNA with a Roche Transcriptor First-Strand cDNA Synthesis Kit (Roche) according to the manufacturer's instructions. Real-time PCR was performed with a Bio-Rad CFX96 system using SYBR Green PCR Master Mix according to the manufacturer's protocol. Relative EGFR mRNA levels were normalized using the housekeeping gene GAPDH. The primers for qRT-PCR were as follows. A forward primer, 5′- CGGGACATAGTCAGCAGTG-3′, and reverse primer, 5′- GCTGGGCACAGATGATTTTG-3′, were used to amplify EGFR. A forward primer 5′-TCAACGACCACTTTGTCAAGCTCA-3′, and reverse primer, 5′- GCTGGTGGTCCAGGGGTCTTACT-3′, were used to amplify GAPDH.

### Chlorhexidine (CHX) assay

Chlorhexidine was added to the culture medium of the cells with a final concentration of 100 ng/ml. Cells were harvested at 0 h, 24 h, 36 h and 48 h. Proteins were extracted and prepared for Western blot analysis.

### *In vivo* tumorigenesis

Animal experiments were performed with the approval of the Peking Union Medical College Animal Care and Use Committees. Ten million tumor cells were resuspended in 0.2 ml phosphate-buffered saline and inoculated into the flanks of 6-week-old, female, athymic nude mice. Five mice were injected in each group. Mice were sacrificed 20 days after inoculation. Tumors were removed, photographed and prepared for Western blot analysis.

### Tissue analyses

The tissue microarray with tumor tissues from 74 cases of CRC was obtained from Shanghai Biochip. Prior patient consent and approval from the Institutional Research Ethics Committee were obtained for the use of these clinical materials for research purposes. The clinical information regarding the samples is summarized in Table 1. The expression of RHBDD1 (1:1000 dilution) and EGFR (1:50 dilution) was detected using immunoperoxidase. Slides were assessed by pathologists blinded to the experimental results and patient outcome. RHBDD1 and EGFR expression was evaluated by an immunostaining score, which was calculated as the sum of the proportion and intensity of the stain. The percentage of positively stained cells was scored on a scale of 0 to 4 as follows: 0 (< 1%), 1 (1–24%), 2 (25–49%), 3 (50–74%), and 4 (75–100%). The staining intensity was scored from 0 to 3 as follows: 0 (negative), 1 (weak), 2 (moderate), and 3 (strong). The scores for percentages of positive cells and staining intensities were then added to generate an immunostaining score for each case. The IS ranged from 0–7. Immunohistochemical scoring was performed without prior knowledge of the clinical response. Immunostained sections were scanned using a microscope (Aperio system).

### Statistical analysis

For qPCR experiments, values represented mean±s.d. of samples measured in triplicate, and each experiment was repeated three times. The significance of differences between experimental groups was analysed using the Student's two-tailed t-test. P<0.05 was considered statistically significant. All the analyses were performed by SPSS and GraphPad Prism 5.0. Correlation analysis were performed using Spearman rank correlation coefficient.

## SUPPLEMENTARY MATERIALS FIGURES


