# Supplementary material for: RHBDD1 upregulates EGFR via the AP-1 pathway in colorectal cancer
Source: Oncotarget. 2017 Feb 25;8(15):25251–60. doi: 10.18632/oncotarget.15694 (PMC5421926; doi:10.18632/oncotarget.15694)
Supplement: Supplementary file 1 [file oncotarget-08-25251-s001.pdf]

RHBDD1 upregulates EGFR via the AP-1 pathway in colorectal cancer

SUPPLEMENTARY MATERIALS

SUPPLEMENTARY FIGURES

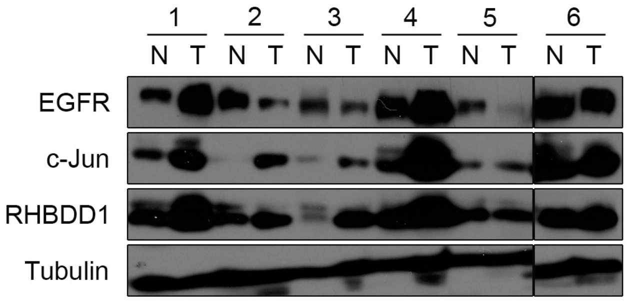

Supplementary Figure 1: RHBDD1, c-Jun and EGFR expression in colorectal cancer. Expression of RHBDD1, c-Jun and EGFR in CRC patients was analyzed by Western blotting using Tubulin as a loading control. T, colorectal tumor samples. N, tumor adjacent tissue.

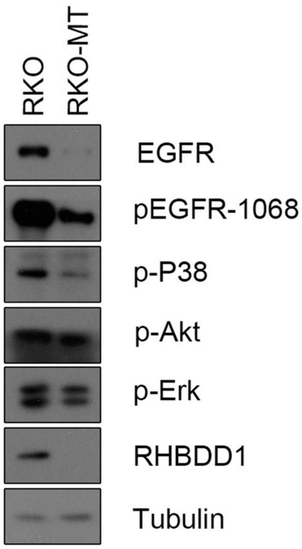

Supplementary Figure 2: RHBDD1 inactivation decreases EGFR downstream signaling pathway protein phosphorylation level.
